# Psychosocial Factors and the Need for Multidisciplinary Support in Nutrition Counselling for Cancer Chemotherapy Patients

**DOI:** 10.3390/nu15122712

**Published:** 2023-06-11

**Authors:** Saori Koshimoto, Tomoko Yamazaki, Koji Amano, Jun Kako, Masako Arimoto, Keiko Saitou, Akiko Hashizume, Takashi Takeuchi, Eisuke Matsushima

**Affiliations:** 1 School of Health Care Sciences, Faculty of Medicine, Tokyo Medical and Dental University, 1-5-45 Yushima, Bunkyo-ku, Tokyo 113-8510, Japan; 2 Faculty of Human Nutrition, Department of Human Nutrition, Tokyo Kasei Gakuin University, 22 Sanban-cho, Chiyoda-ku, Tokyo 102-8341, Japan; 3 Graduate School of Health Care Sciences, Tokyo Medical and Dental University, 1-5-45 Yusima, Bunkyo-ku, Tokyo 113-8510, Japan; 4 Palliative and Supportive Care Center, Osaka University Hospital, 2-15 Yamadaoka, Suita 565-0871, Osaka, Japan; 5 Graduate School of Medicine, Mie University, 2-174 Edobashi, Tsu 514-08507, Mie, Japan; 6 Department of Clinical Nutrition, Tokyo Medical and Dental University Hospital, 1-5-45 Yushima, Bunkyo-ku, Tokyo 113-8510, Japan; 7 Department of Nursing, Tokyo Medical and Dental University Hospital, 1-5-45 Yushima, Bunkyo-ku, Tokyo 113-8510, Japan; 8 Liaison Psychiatry and Psycho-Oncology Unit, Department of Psychiatry and Behavioral Sciences, Graduate School of Medical and Dental Sciences, Tokyo Medical and Dental University, 1-5-45 Yushima, Bunkyo-ku, Tokyo 113-8510, Japan

**Keywords:** cancer, chemotherapy, nutrition counselling, psychosocial factors, multidisciplinary support, quality of life

## Abstract

This study aims to identify the background factors and experiences of patients with cancer with eating-related problems who require nutrition counselling. Using a mixed-methods approach, this secondary analysis study was conducted on patients with head and neck, oesophageal, gastric, colorectal, or lung cancers who were receiving outpatient chemotherapy. They completed a questionnaire measuring nutrition impact symptoms, eating-related distress, and quality of life (QOL). Patients who required nutrition counselling were interviewed to identify the specific issues they experienced. We reported on nutritional status and nutrition impact symptoms in a previous study. Of the 151 participants, 42 required nutrition counselling. Background factors associated with nutrition counselling were related to the following psychosocial variables: small number of people in the household, undergoing treatment while working, low QOL, and eating-related distress. Four themes were extracted from the specific issues experienced by patients: motivation for self-management, distress from symptoms, seeking understanding and sympathy, and anxiety and confusion. The desire for nutrition counselling was attributable to ‘anxiety caused by the symptoms’ and ‘confusion about the information on eating’. Healthcare professionals should promote multidisciplinary collaboration after considering the factors associated with the required nutrition counselling to provide nutritional support.

## 1. Introduction

Chemotherapy causes nutrition impact symptoms (NIS), such as taste disorder, olfactory disorder, and constipation [1,2], and can aggravate pre-existing nutritional issues [3,4]. Studies have shown that 50–80% of patients with advanced cancer experience cachexia, a systemic metabolic disorder characterized by weight loss and loss of skeletal muscle mass, which affects their quality of life (QOL) [5]. Approximately 80% of patients with cancer experience anorexia and weight loss [6]. Moreover, numerous patients with advanced cancer and their family members suffer from eating-related distress (ERD) [7]. Nutritional support has been shown to reduce mortality and improve the QOL of patients with cancer [8]. Preliminary research shows that 77.5% of outpatients receiving chemotherapy need nutrition counselling, the need for which is associated with their QOL [9]. The European Society for Clinical Nutrition and Metabolism recommends nutritional intervention to increase oral intake in cancer patients who can eat but are malnourished or at risk of malnutrition [1]. The provision of clinically assisted nutrition in patients with advanced cancer is individually managed based on evidence-based guidance [10]. Nutritional intervention comprises dietary advice and treatment of symptoms that impair food intake and may include the prescription of oral nutritional supplements or tube feeding [11]; however, nutritional intervention alone does not address psychosocial issues.

In addition to nutritional intervention, some patients may require nutrition counselling, which is not limited to ensuring adequate caloric and protein intake. Diet is related to various contextual factors that affect patients, such as anxiety regarding weight loss [12], building physical strength to fight the disease [13], and the desire to maintain pre-illness lifestyles [14] as much as possible. Therefore, it is imperative to understand the psychosocial factors of patients who require nutritional intervention, in addition to their physical factors. While some studies have explored the views of cancer patients in relation to nutrition support using a mixed-method approach and others have examined specific circumstances in which patients require counselling [15,16], to the best of our knowledge, no study has investigated psychosocial factors related to nutrition counselling. Thus, the two main objectives of the study were to: (i) identify the background factors of patients with cancer receiving chemotherapy who required nutrition counselling, and (ii) elucidate specific eating-related problems that these patients wished to discuss in counselling.

## 2. Materials and Methods

### 2.1. Participants

This is a secondary analysis of a survey study on the needs of cancer patients undergoing outpatient chemotherapy for nutritional counselling [9]. The survey was conducted between August 2016 and November 2017, when patients visited an urban university hospital for chemotherapy in Japan. The investigator waited in the chemotherapy room for seven hours a day. Subsequently, they requested all patients who received chemotherapy as outpatients at the hospital and met the eligibility criteria to participate in the study. Those who were willing to participate were included in the survey. The participant inclusion criteria were: (i) aged 20–80 years with a head and neck, oesophageal, stomach, colorectal, or lung cancer diagnosis, who had never received nutrition counselling during chemotherapy; (ii) undergoing outpatient chemoradiotherapy, adjuvant chemotherapy, palliative chemotherapy, or targeted treatment; and (iii) willing to participate in the research and provide consent. Patients with severe dementia/mental disorders and those deemed by a doctor as unable to participate owing to psychological/physical reasons were excluded. The patients were given the questionnaire during chemotherapy, which lasted for three to seven hours; this provided sufficient time to complete the survey.

### 2.2. Ethical Considerations

The Institutional Review Board of the Medical Research Ethics Committee of Tokyo Medical and Dental University (no. M2015-578) approved this study. The survey began after registering this study as a clinical trial (UMIN registration no. 000021540). Written informed consent was obtained from all the participants.

### 2.3. Study Design

In this study, we qualitatively analyzed patients’ expressed preferences for nutrition counselling to identify the specific topics they wished to discuss. In our previous investigation, we took anthropometric measurements of the participants (body mass index [BMI], muscle mass, body fat percentage, estimated bone mass, and body water content) using bioelectrical impedance analysis. Then, participants completed a questionnaire to assess NIS, QOL, and ERD. Their albumin level, C-reactive protein level, and modified Glasgow Prognostic Score (mGPS, a measure of cachexia based on the sum of albumin and C-reactive protein levels) were determined based on their medical records. While our previous study reported associations with QOL, nutritional status, and ERD [9], the content of the interview has not been reported. This secondary analysis was based on a qualitative analysis of the content of interviews with patients, summarized using a mixed-methods approach.

Patients who answered “yes” to the question “Do you want nutrition counselling?” were included in the study and interviewed to determine what specific issues they would like to discuss. Two individual nutritional consultations with a registered dietitian specializing in cancer were scheduled at one-month intervals, which is the standard in Japan. All surveys, measurements, interviews, and nutrition counselling sessions were scheduled for the same days the participants had outpatient chemotherapy. During the nutrition counselling, the patients’ food intake frequency and activities of daily living were assessed, and advice was given on food quantity, nutritional quality, energy balance, and how to manage the side effects of chemotherapy.

### 2.4. Questionnaires

The Patient-Generated Subjective Global Assessment Short Form (PG-SGA SF) was used to measure NIS [17]. The PG-SGA SF scores were calculated based on its manual and a high score indicated more severe nutritional problems. Each item on the PG-SGA SF questionnaire covers patients with weight loss, anorexia, nausea, constipation, diarrhea, thirst, dysgeusia, olfactory disorder, vomiting, dysphagia, early satiety, fatigue, or pain.

Patients’ experience of ERD was assessed using the ERD questionnaire [18] comprising 19 items on distress originating from the patients’ own feelings (ERD-1), concerns regarding information about the patients’ diet (ERD-2), and the relationship between patients and their family members (ERD-3). The participants’ responses were based on a 4-point Likert-type scale ranging from 1 (‘No’) to 4 (‘Always’) (Table A1).

QOL was measured using the European Organization for Research and Treatment of Cancer Quality of Life Questionnaire (EORTC-QLQ) C30 [19]. This questionnaire measures the global health domain and the physical functioning domain; the items were role functioning, emotional functioning, cognitive functioning, social functioning, and symptom scales. A high score on the functional scale represents a high/healthy level of functioning, a high score on the global health status/QOL scale represents a high QOL, and a high score on the symptom scale/item represents a high level of symptomatology/problems.

Social factors were determined through questions regarding education level, marital status, the number of people in the household, employment status, monthly food expenses, and information about who prepares meals.

### 2.5. Interviews

Semi-structured interviews were conducted with participants who required nutrition counselling. They were asked the following questions: (i) Do you think nutrition counselling is necessary for chemotherapy treatment? (ii) Do you require nutrition counselling from a dietician now? (iii) What do you wish to discuss during nutrition counselling? The interview questions were designed to elicit descriptive responses from the participants. The interviewer transcribed participants’ responses on a survey form. To interpret the concepts behind the answers, they were encoded and categorized using Krippendorff’s content analysis method [20]. Content analysis views data as representations, not of physical events but of texts and expressions that are created to be seen, read, interpreted, and acted on based on their meaning.

### 2.6. Statistical Analysis

Background factors influencing nutrition counselling were analyzed using logistic regression analysis (maximum likelihood estimation). The dependent variable was whether the participant required nutrition counselling. The independent variables were age, QOL, PG-SGA SF score, employment status (yes vs. no), number of people in the household (two or fewer vs. three or more), meal preparation (self vs. someone else), food expenses (25,000 yen)/month or more vs. under 25,000 yen (in Japan, the mean cost of food per person is approximately 25,000 yen), and ERD 1–3 score. SPSS version 25 (IBM, Armonk, NY, USA) was used, and statistical significance was set at 5%.

For the interview data, content analysis was conducted based on Krippendorff’s guidelines [20]. The interview content was encoded, and statements with similar meanings were organized into conceptual categories. To ensure reliability, content analysis was performed independently by two researchers who had experience in content research and clinical experience in the field of palliative care. Two palliative care specialists supervised the analysis. The qualitative analysis results were integrated with quantitative data as a mixed-methods design [15,16].

## 3. Results

### 3.1. Patient Characteristics

**Figure 1:**
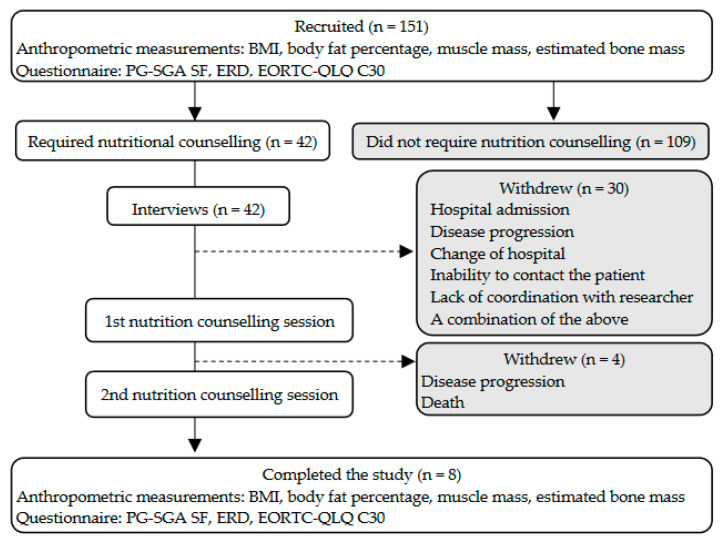
Research plan and participants’ BMI, body mass index; EORTC-QLQ C30, European Organization for Research and Treatment of Cancer Quality of Life Questionnaire Version 3.0; ERD, eating-related distress; PG-SGA SF, Patient-Generated Subjective Global Assessment Short Form.

Figure 1 shows the overall research plan. The present qualitative analysis was conducted on the data obtained from the interviews with patients. Table 1 illustrates the patients’ characteristics. Of the 151 patients, 42 (27.8%) required nutrition counselling. There was no significant effect of sex, performance status, cancer site, or BMI on whether patients required nutrition counselling. Of the 42 patients who required nutrition counselling, only 8 (19.0%) completed both sessions. Patients dropped out of the study owing to death, transfer to another hospital, deterioration of their medical condition, loss of contact, and lack of coordination with the researcher.

This secondary analysis reports the content and background factors obtained through the interviews with 42 patients, which were conducted prior to the nutritional counselling.

### 3.2. Psychosocial Factors That Influenced Patients’ Requirements for Nutrition Counselling

Table 2 illustrates the factors that influenced the nutrition counselling requirements of 42 patients using logistic regression analysis. The adjusted odds ratio (OR) was calculated with a 95% confidence interval (CI). The coefficient omnibus test model assured the significance of the regression equation (*p* < 0.001). Hosmer–Lemeshow test significance was *p* = 0.178, indicating that the prediction accuracy was high. The discrimination accuracy of this logistic regression analysis was 78.7%. Sex, performance status, cancer site, disease stage, BMI, albumin level, C-reactive protein level, mGPS, and individual symptoms were not significantly associated with patients’ nutrition counselling requirement. However, having few members in the household (*p* = 0.014), working while undergoing treatment (*p* = 0.015), having low QOL (*p* = 0.023), and experiencing distress owing to concerns about diet information (ERD-2 score, *p* = 0.036) were all significantly associated with patients requiring nutrition counselling. The education level and marital status questionnaire items could not be analyzed owing to the large number of unanswered questions pertaining to these items.

### 3.3. Specific Issues That Led to a Desire for Nutrition Counselling

Table 3 shows the specific issues that 24 participants wished to discuss during nutrition counselling from experiences of eating-related problems. The content analysis revealed the following conceptual categories:

(1) Motivation for self-management: The participants regarded information about nutrition and diet positively, relating it to their treatment and physical condition.
(2) Distress from symptoms: The participants struggled with nutrition and diet as they dealt with NIS, side effects of chemotherapy, and complications related to other symptoms.
(3) Seeking understanding and sympathy: The participants felt that people around them needed to understand that they were trying to manage food intake levels and NIS.
(4) Anxiety and confusion: The participants were confused and swayed by unclear information owing to their fear of not being able to eat.

Among the participants who took part in nutrition counselling, ‘distress from symptoms’ and ‘information regarding nutritional balance’ were the most common topics they wanted to discuss.

## 4. Discussion

This study identified the psychosocial factors related to patients with cancer who require nutrition counselling and the specific issues they wished to discuss from their experiences of eating-related problems. The results revealed that the patients believed that diet helps relieve NIS and improve physical health. In contrast, they experienced nutrition-related distress owing to NIS and did not have enough reliable information about nutrition. This led to anxiety about the side effects of chemotherapy and confusion about how to deal with the NIS. Therefore, individual nutrition counselling and specialist information should be provided to patients to help combat nutrition-related distress and confusion [21]. Some studies suggest that dietitians may serve to protect patients against potentially harmful dietary supplement use, fad diets, and other unproven or extreme diets. It is reported that up to 48% of patients with cancer pursue, or are interested in, ‘fad’ or popular diets [22]. The existing literature has shown that nutrition support has the potential to reduce nutritional risk during cancer treatment and address eating problems, and optimal management could improve treatment tolerance and decrease treatment interruptions. Previous reports are consistent with the findings of our study; for example, Hopkinson reported that support for self-management of nutritional risk may protect against malnutrition and be an important aspect of multimodal therapies to arrest the progression of cachexia [23]. Additionally, Arends established that therapeutic options comprise counselling, oral nutritional supplements, enteral and parenteral nutrition, metabolic modulation [21], exercise training, and supportive care to enable and improve the intake of adequate amounts of food, and psycho-oncology and social support [1]. Cancer patients who are affected psychosocially due to reduced eating ability need care from professionals [24].

This study showed that most patients who required nutrition counselling lived alone or with one other person, continued working while undergoing chemotherapy treatment, and had a low QOL (Table 2). Additionally, previous studies have reported a link between poor appetite, anxiety, depression, and loneliness following cancer therapy [25,26]. The results of these studies imply that patients might face difficulties in managing both work and treatment and might want their colleagues and dieticians to understand their symptoms and empathize with their efforts. Alternatively, patients may want to confirm correct information and eliminate anxiety. Ultimately, the desire for nutrition counselling emerged more because of the influence of ‘anxiety caused by the symptoms’ and ‘confusion about information on eating’. Since this study highlighted patients’ state of confusion caused by being overwhelmed with unclear information about food, the impact of dietary information spread through social networks should be further investigated. Online sources reported a conceptual framework of cancer-related nutrition misinformation [27]. Socializing and gathering at mealtimes play an important role in maintaining a psychological balance that contributes to overall QOL and is closely associated with social contact [28]. Therefore, it is imperative to provide psychosocial support for patients who require nutrition counselling. As shown in Table 3, if a patient is unable to eat because of anxiety over future events, specific ways to deal with NIS should be recommended to them. Furthermore, healthcare professionals should consider their issues related to family relationships and help nurture sustained feelings of hope and well-being. Additionally, cancer patients undergoing chemotherapy should be encouraged to share valuable moments with friends and family to reduce diet-associated psychosocial distress [29,30]. It is important to empathize with patients’ anxiety about lack of appetite and weight loss and help manage their feelings of remorse for not being able to meet family expectations. This attitude seems to change during the patients’ illness trajectory. Previous findings indicated that patients’ eating deficiencies caused hesitancy about attending social gatherings and led to complete avoidance of such situations. This made them feel lonely and separated from the life they had lived as healthy people [30].

The results of this study show that the need for nutrition counselling is related to psychosocial factors such as living situation, employment status, and psychosocial outcomes, including anxiety and confusion regarding NIS and diet. Nutritional support for patients undergoing cancer chemotherapy requires nutritionists to collaborate with attending physicians, psycho-oncologists, nurses, pharmacists, clinical psychologists, social workers, and physical therapists while sharing relevant information with each other and the patients. Therefore, a multidimensional approach is recommended to support individuals experiencing nutritional issues [21]. Based on this study’s results, we recommend that nutritional screening to determine nutritional status should incorporate psychosocial assessment for ERD and other nutritional issues in addition to conventional assessments, including symptoms and blood test findings. Nutrition counselling is typically conducted by a dietician, but if psychosocial problems are identified, counselling sessions should be set up with a psychologist, a nurse specializing in cancer care, or a psycho-oncologist in addition to a dietician. Furthermore, dietitians should develop skills related to psycho-oncology, particularly related to an understanding that patients often experience anxiety and desperation about not being able or not knowing what to eat. Thus, combination therapy and multimodal care are likely to be critical in nutrition counselling [22,31,32,33,34].

**Figure 2:**
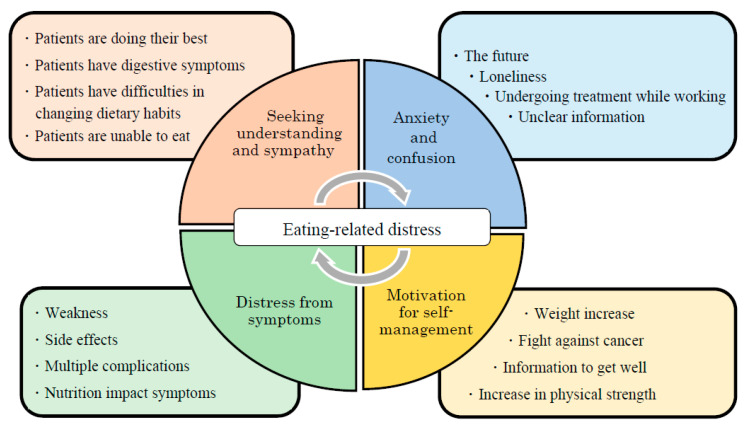
Themes of psychosocial and physical support associated with eating-related distress.

Many patients with advanced cancer undergoing chemotherapy are anxious about their future. Figure 2 illustrates themes of psychosocial and physical support associated with ERD. Therefore, as part of a holistic treatment approach, dieticians should collaborate with specialists in relevant fields to develop multidisciplinary interventions. Furthermore, rather than merely insisting that patients follow their instructions, medical professionals should consider both the symptoms and emotions of patients. Future studies could explore patients’ involvement in decision-making processes regarding what nutritional treatment to choose and how they can adhere to such treatment. A patient may want to adhere to nutritional treatment but be unable to do so because of a debilitating condition. Given such eventualities, the medical staff should ensure that nutrition interventions consider such information and support patients accordingly.

Based on objective assessments combining NIS, nutritional status, and other relevant data, the qualitative summary of patients’ statements included in this study helped identify their experiences and circumstances. Thus, to develop realistic nutritional support, dieticians should perform assessments from a multifaceted perspective as part of nutrition counselling. Furthermore, to assess the real situation in clinical practice, it is useful to employ a mixed-methods design that combines qualitative assessments of the patients’ subjective data and a quantitative analysis to objectively evaluate their conditions. 

This study had the following limitations. First, only eight participants completed the two nutrition counselling sessions. High dropout rates are consistent with advanced cancer, and the American Society of Clinical Oncology guidelines stated that many studies reported high rates of patient dropout, and the difficulty of interventional studies for patients with advanced cancer is a common issue in the field [22]. Future studies should include a larger and more representative sample to better reflect the population. Second, this study has limited generalizability, as all the participants were recruited from one hospital and one nationality. Therefore, future studies should be conducted in other hospitals (e.g., in rural locations, in different countries) to examine whether different participant groups yield similar results. Further cohort studies are needed to investigate the impact of improved nutritional status on survival, chemotherapy side effects, and motivation for treatment, and to determine what type of nutrition counselling is effective.

## 5. Conclusions

This study elucidated the psychosocial factors of patients who require nutrition counselling and the specific issues they wished to discuss. The results showed that patients who required nutrition counselling tended to live with fewer people, had low QOL, and were working during treatment. They often experienced distress originating from concerns regarding their diet. Four themes were extracted using content analysis regarding the specific issues that patients who require nutrition counselling wished to discuss: (i) motivation for self-management, (ii) distress from symptoms, (iii) seeking understanding and sympathy, and (iv) anxiety and confusion. Ultimately, the desire for nutrition counselling emerged mostly because of the influence of ‘anxiety caused by the symptoms’ and ‘confusion about information on eating’. Therefore, multidisciplinary support related to nutritional symptoms, and psychological (anxiety, confusion) and social (household structure, employment situation) factors may help cancer patients undergoing outpatient chemotherapy to enhance their QOL.

## Figures and Tables

**Table 1:** Characteristics of patients who required nutrition counselling.

Variable	Required Nutrition Counselling (*n* = 42)	Did Not Require Nutrition Counselling (*n* = 109)
age (years) *	65.5 ± 9.1	66.9 ± 8.8
female *n* (%)	11 (26.2)	41 (37.6)
male *n* (%)	31 (73.8)	68 (62.4)
PS (median)	2	2
cancer site *n* (%)		
head and neck	2 (4.8)	13 (11.9)
oesophageal	5 (11.9)	7 (6.4)
gastric	4 (9.5)	18 (16.5)
colorectal	17 (40.5)	32 (29.4)
lung	14 (33.3)	39 (35.8)
disease stage (median)	III	III
BMI kg/(cm)^2^ *	21.1 ± 3.6	21.8 ± 4.1
serum albumin g/dL *	3.8 ± 0.4	3.5 ± 1.0
C-reactive protein mg/dl *	0.8 ± 1.2	0.7 ± 1.3
mGPS (median)	2	2
symptoms *n* (%)		
anorexia	19 (45.2)	43 (39.4)
weight loss of 2% or more	9 (21.4)	13 (11.9)
constipation	16 (38.1)	27 (24.8)
diarrhea	7 (16.7)	23 (21.1)
thirst	9 (24.4)	21 (19.3)
dysgeusia	20 (47.6)	40 (36.7)
olfactory disorder	11 (26.2)	15 (13.8)
vomiting	10 (23.8)	21 (19.3)
dysphagia	8 (19.0)	21 (19.3)
early satiety	9 (21.4)	29 (26.6)
fatigue	22 (52.4)	51 (46.8)
pain	15 (35.7)	21 (19.3)
PG-SGA SF *	8.6 ± 5.0	7.1 ± 5.1
QOL score (EORTC-QLQ C30) *		
global health status	52.8 ± 16.0	60.2 ± 18.8
physical functioning	75.4 ± 22.7	79.0 ± 18.1
role functioning	76.6 ± 24.7	77.5 ± 28.0
emotional functioning	80.2 ± 18.1	87.1 ± 15.7
cognitive functioning	82.5 ± 19.6	90.6 ± 15.0
social functioning	72.4 ± 26.0	82.1 ± 22.7

BMI, body mass index; EORTC-QLQ C30, European Organization for Research and Treatment of Cancer Quality of Life Questionnaire; mGPS, modified Glasgow Prognostic Score; PG-SGA SF, Patient-Generated Subjective Global Assessment–Short Form; PS, performance status; QOL, quality of life. * Mean ± standard deviation.

**Table 2:** Psychosocial factors that influenced nutrition counselling requirements.

Independent Variables	Required Nutrition Counselling (*n* = 42)		Did Not Require Nutrition Counselling (*n* = 109)		*p*-Value
Number of people in the household (one or two) *n* (%)	33 (80.5)		62 (57.4)		0.009
Employment status (working) *n* (%)	18 (42.9)		34 (31.8)		0.203
Global health status on QOL * (mean ± SD)	52.8 ± 16.0		60.3 ± 18.8		0.028
ERD-2 ^†^ (mean ± SD)	2.4 ± 0.6		2.1 ± 0.5		0.002
Independent variables	Partial regression coefficient	Odds ratio	95% confidence interval		*p*-value
			min.	max.	
Number of people in the household	1.39	4.00	1.33	12.02	0.014 *
Employment status	−1.14	0.32	0.13	0.80	0.015 *
Global health status on QOL *	−0.03	0.97	0.95	1.00	0.023 *
Distress from ERD treatment ^†^	0.93	2.52	1.06	6.00	0.036 *

ERD, eating-related distress; QOL, quality of life; SD, standard deviation. * QOL scored using the European Organization for Research and Treatment of Cancer Quality of Life Questionnaire. ^†^ ERD-2 (the distress originating from concerns regarding information about the patient’s diet) calculated by the mean score (1 = strongly disagree, 2 = disagree, 3 = agree, 4 = strongly agree). * *p* < 0.05.

**Table 3:** Specific issues patients desired to discuss during nutrition counselling (*n* = 42).

Category	Sub-Categories (*n*)	Codes (*n*)
Motivation for self-management	Information regarding nutritional balance (11)	I want to know about the absorption of fats and nutrients, and meal combinations. (2)
		I want advice about specific foods. (3)
		I want to know more about nutrients. (1)
		I want to know more about quality/quantity and nutrition. (2)
		I want informational materials about the classification of nutrients. (1)
		What meals have a high nutrient content and provide a good nutritional balance? (1)
		Are my portion sizes large enough? (1)
	Information on how to adjust diet to improve physical condition (6)	Which foods are easy to digest? (2)
		Which foods increase physical strength? (1)
		How can I increase my weight? No matter what I do, my weight does not seem to increase. (1)
		What can I eat and is easy for me to eat? (1)
		I have heard that massaging the salivary gland is good. Please teach me how to do it. (1)
	Information regarding foods beneficial for treatment (7)	What foods can I eat despite having cancer? (2)
		What foods should I not eat owing to cancer? (1)
		Which foods are effective against cancer? (2)
		How can I adjust my foods, nutrition, and meals according to my type of cancer? (1)
		Which foods can help with the treatment without supplements? (1)
Distress from symptoms	Distress from nutrition impact symptoms and side effects (12)	I do not know about suitable menus during chemotherapy. (1)
		I do not know how to manage medication with food (ingredients). (1)
		Which cooking methods are suitable for my symptoms? (1)
		How can I deal with the smell of certain foods? (1)
		It is difficult to improve my anaemia. (1)
		I worry that chemotherapy will harm the function of my kidneys and liver. What should I eat to prevent this? (1)
		How can I supplement my nutrition when I cannot eat owing to mouth ulcers? (1)
		How can I deal with taste disorders? (1)
		It is difficult to control constipation. (1)
		What can I eat when my condition worsens owing to side effects? (1)
		It is difficult to control medication timings when I cannot eat owing to side effects. (2)
	Difficulty in handling multiple complications (6)	It is difficult to coordinate diabetes and cancer treatments. (4)
		I have both hypertension and diabetes; therefore, I am confused over which treatment to prioritize. (1)
		What can I eat when I have intestinal obstruction? (1)
Seeking understanding and sympathy	Eagerness for others to understand their inability to eat (10)	I am doing my best but cannot eat because my throat hurts. (1)
		I want you to know that it is difficult to prepare meals because of severe numbness. (1)
		You may not realize it, but I cannot eat because of my mouth ulcers. (1)
		I know I must eat, but it is difficult owing to perleche and taste disorder. (1)
		I want to eat, but when I do, the food gets stuck in my throat, and I cannot eat enough. (1)
		I had nutrition counselling in the past, but I could not do all the things I was advised. (1)
		I understand that I should eat what I can when I want to eat, but I cannot eat. (2)
	Desire for others to understand their symptoms (3)	I am worried about having diarrhea at work. (2)
		I want my spouse to understand the changes in my sense of taste and provide foods that are easy to eat. (1)
Anxiety and confusion	Anxiety about being unable to eat in the future (8)	What should I do if I cannot eat? (4)
		What will happen in the future? (2)
		I would like advice regarding losing weight if the need arises. (2)
	The burden of changing dietary habit (5)	I always eat the same things. (1)
		I have no appetite, but I can drink alcoholic beverages. (1)
		It is difficult to find foods I can and should eat. (1)
		I am struggling with my dislike of vegetables. (1)
		I am struggling with being a picky eater. (1)
	Swayed by unclear information about foods (6)	Can I eat meat, eel, or purified rice? I heard these foods are bad. (1)
		Are carrots, juice made from green leafy vegetables, and hydrogen water effective? Is there any scientific basis for this? (1)
		I cannot eat vegetables, but is it okay to drink juice made from green leafy vegetables? (1)
		I read a cookbook by Dr. A of a certain university, which said that I should prepare meals with brown rice, vegetables, and fish. However, I lost weight when I followed that advice. (1)
		I heard that unpolished rice is good against cancer, but I lost weight when I followed this advice. (1)
		Does a low-carbohydrate diet inhibit the growth of cancer cells? (1)
	Fixated on dietary rules (5)	I am worried about getting an infection when I take off my mask to eat. (1)
		I was told to avoid raw foods in post-operative counselling, and so I have avoided them for years. (1)
		I was told to avoid brown rice and seaweed in post-operative counselling, and so I have avoided them for years. (1)
		I use steroids to improve my appetite; can I take them with milk to protect my stomach? (1)
		I suck on candy to reduce the side effects, but my family says that I have no appetite because of this. Does candy suppress one’s appetite? (1)

## Data Availability

The data that support the findings of this study are available from the corresponding author upon reasonable request.

## Appendix A

**Table A1:** Questionnaire items of eating-related distress (ERD) for cancer patients receiving chemotherapy.

Question Items	Likert Scale			
Distress originating from the feelings of patients themselves (ERD-1)	No	Sometimes	Frequently	Always
I feel that lack of nutrition makes my condition worse.	1	2	3	4
I think that I cannot eat because of a lack of effort on my part.	1	2	3	4
I feel that it is the natural course of the disease that I cannot get enough nutrition and that I lose weight.	1	2	3	4
I am disappointed to find that I cannot eat enough.	1	2	3	4
I feel that I should make efforts to get enough nutrition even if I have a bad physical condition.	1	2	3	4
Distress originating from concerns regarding information about the patient’s diet (ERD-2)	No	Sometimes	Frequently	Always
I think that losing weight results from a lack of nutrition and that I can gain weight if I get enough nutrition.	1	2	3	4
I have eaten what I want without consideration of calories and nutritional composition.	1	2	3	4
I have made myself concerned about my daily diet.	1	2	3	4
I have tried to eat various foods.	1	2	3	4
I have tried to eat a high-calorie and well-balanced diet.	1	2	3	4
I have found it useless to consult medical staff about my daily diet.	1	2	3	4
I would like to consult an expert who has specific knowledge of nutrition therapy.	1	2	3	4
Distress originating from the relationship between patients and their families (ERD-3)	No	Sometimes	Frequently	Always
I am burdened by meals that are made for me with kindness.	1	2	3	4
I often experience conflict about meals when a person makes them for me.	1	2	3	4
I feel that I disregard the kindness of the person who makes meals for me when I cannot eat.	1	2	3	4
I try to have a good meal not for myself but for family members.	1	2	3	4
I avoid talking about food and eating with family members.	1	2	3	4
Although family members and friends recommend various foods to me, I am just confused.	1	2	3	4
I often feel that I am forced to eat.	1	2	3	4

Participants responded on a four-point Likert-type scale, ranging from 1 (no) to 4 (always).

**Table A2:** Comparison of characteristics before and after nutrition counselling (*n* = 8).

Variable	Nutrition Counselling		*p* Value ^§^
	Before	After	
	Mean ± SD	Mean ± SD	
Age	70.6 ± 5.9	70.6 ± 5.9	-
Cancer Stage (median)	III	III	-
BMI (kg/m^2^)	20.6 ± 2.1	20.5 ± 1.9	0.833
Alb (g/dL)	3.9 ± 0.4	3.6 ± 0.2	0.018
CRP (mg/dL)	0.4 ± 0.4	0.9 ± 1.0	0.063
mGPS	0.4 ± 0.5	0.5 ± 0.5	0.317
PG-SGA SF Score	7.3 ± 4.0	7.4 ± 4.3	1.000
QOL (EORTC-QLQ C30)			
Global Health Status	58.3 ± 13.4	59.4 ± 21.5	0.854
Physical Functioning	82.5 ± 10.0	85.0 ± 7.8	0.461
Role Functioning	77.1 ± 21.7	87.5 ± 19.4	0.102
Emotional Functioning	85.4 ± 13.9	88.6 ± 11.7	0.581
Cognitive Functioning	91.7 ± 17.8	91.7 ± 17.8	1.000
Social Functioning	83.3 ± 21.8	91.7 ± 17.8	0.180
Dyspnoea	37.5 ± 21.4	29.1 ± 11.8	0.157
Sleep Disorder	16.7 ± 17.8	16.7 ± 25.2	0.705
Poor Appetite	29.2 ± 27.8	29.2 ± 27.8	1.000
Diarrhea	16.7 ± 35.4	12.5 ± 35.4	0.655
Constipation	20.8 ± 30.9	20.8 ± 24.8	1.000
Pain	20.8 ± 14.8	16.7 ± 17.8	0.715
Fatigue	47.2 ± 26.4	29.2 ± 23.0	0.042
Nausea/Vomiting	4.2 ± 7.7	2.1 ± 5.9	0.317
Financial Problems	12.5 ± 24.8	4.2 ± 11.8	0.180

Alb, serum albumin; BMI, body mass index; CRP, C-reactive protein; EORTC-QLQ C30, European Organization for Research and Treatment of Cancer Quality of Life Questionnaire Version 3.0; mGPS, modified Glasgow Prognostic Score; PG-SGA-SF, Patient-Generated Subjective Global Assessment-Short Form; QOL, quality of life; and SD, standard deviation. ^§^ Wilcoxon signed-rank test.

**Table A3:** Relationship between eating-related distress and nutrition counselling (*n* = 8).

Question Items	Nutrition Counselling		
Distress originating from the feelings of patients themselves (ERD-1)	before ^†^	after ^†^	*p* value ^§^
I feel that lack of nutrition makes my condition worse.	2.0	2.0	1.000
I think that I cannot eat because of a lack of effort on my part.	1.3	1.5	0.157
I feel that it is the natural course of the disease that I cannot get enough nutrition and that I lose weight.	1.7	1.7	1.000
I am disappointed to find that I cannot eat enough.	1.9	1.9	1.000
I feel that I should make efforts to get enough nutrition even if I have a bad physical condition.	2.4	2.1	0.680
Distress originating from concerns regarding information about the patient’s diet (ERD-2)	before ^†^	after ^†^	*p* value ^§^
I think that losing weight results from a lack of nutrition and that I can gain weight if I get enough nutrition.	1.9	2.4	0.357
I have eaten what I want without consideration of calories and nutritional composition.	1.4	2.0	0.180
I have made myself concerned about my daily diet.	3.4	2.5	0.102
I have tried to eat various foods.	3.9	3.8	0.317
I have tried to eat a high-calorie and well-balanced diet.	3.4	3.4	1.000
I have found it useless to consult medical staff about my daily diet.	1.1	1.0	0.317
I would like to consult an expert who has specific knowledge on nutrition therapy.	2.9	2.4	0.336
Distress originating from the relationship between patients and their families (ERD-3)	before ^†^	after ^†^	*p* value ^§^
I am burdened by meals that are made for me with kindness.	1.2	1.1	0.317
I often experience conflict about meals when a person makes them for me.	1.6	1.8	0.564
I feel that I disregard the kindness of the person who makes meals for me when I cannot eat.	1.6	1.6	1.000
I try to have a good meal not for myself but for family members.	1.5	1.5	1.000
I avoid talking about food and eating with family members.	1.1	1.0	0.317
Although family members and friends recommend various foods to me, I am just confused.	2.1	1.5	0.025
I often feel that I am forced to eat.	1.4	1.6	0.317

ERD, eating-related distress. ^†^ Mean values: 1 = no, 2 = sometimes, 3 = frequently, 4 = always. ^§^ Wilcoxon signed-rank test.
